# Familial Dysalbuminemic Hyperthyroxinemia: An Underdiagnosed Entity

**DOI:** 10.3390/jcm9072105

**Published:** 2020-07-03

**Authors:** Xavier Dieu, Nathalie Bouzamondo, Claire Briet, Frédéric Illouz, Valérie Moal, Florence Boux de Casson, Natacha Bouhours-Nouet, Pascal Reynier, Régis Coutant, Patrice Rodien, Delphine Mirebeau-Prunier

**Affiliations:** 1 Laboratoire de Biochimie et Biologie Moléculaire, CHU Angers, 4 rue Larrey, CEDEX 9, 49933 Angers, France; NaBouzamondo@chu-angers.fr (N.B.); VaMoal@chu-angers.fr (V.M.); FlBouxDeCasson@chu-angers.fr (F.B.d.C.); PaReynier@chu-angers.fr (P.R.); DePrunier@chu-angers.fr (D.M.-P.); 2 UMR CNRS 6015-INSERM U1083, 3 rue Roger Amsler, 49100 Angers, France; Claire.Briet@chu-angers.fr (C.B.); PaRodien@chu-angers.fr (P.R.); 3 Centre de référence des maladies rares de la thyroïde et des récepteurs hormonaux, CHU Angers, 4 rue Larrey, CEDEX 9,49933 Angers, France; FrIllouz@chu-angers.fr (F.I.); NaBouhours-Nouet@chu-angers.fr (N.B.-N.); ReCoutant@chu-angers.fr (R.C.); 4 Service d’Endocrinologie-Diabétologie-Nutrition, CHU Angers, 4 rue Larrey, CEDEX 9, 49933 Angers, France; 5 Service d’Endocrinologie et Diabétologie Pédiatrique, CHU Angers, 4 rue Larrey, CEDEX 9, 49933 Angers, France

**Keywords:** resistance to thyroid hormone, immunoassay interference, THRB, albumin, familial dysalbuminemic hyperthyroxinemia

## Abstract

Resistance to thyroid hormone (RTH) is a syndrome characterized by impaired sensitivity of tissues to thyroid hormone (TH). The alteration of TH-binding proteins, such as in Familial Dysalbuminemic Hyperthyroxinemia (FDH), can mimic the abnormal serum thyroid tests typical of RTH. We aimed to characterize a population referred to our center with suspected RTH and estimate the proportion of patients with FDH. For 303 different families, we collected clinical and hormonal data and sequenced the thyroid hormone receptor β gene (*THRB*) and exon 7 of the albumin gene (*ALB*). We found 56 *THRB* variants (i.e., 38% of the 303 index cases, called RTHβ group). Among the samples screened for FDH variants, 18% had the variant R218H in *ALB* (FDH group); in addition, 71% of the cases had neither variant (non-FDH/RTHβ group). Patients with FDH had significantly lower free T3 (fT3) and free T4 (fT4) levels and more often an isolated elevation of fT4 than RTHβ patients. Clinically, patients with FDH had fewer symptoms than patients with RTHβ. Our study suggests that FDH should be systematically considered when examining patients suspected of having RTH. In most cases, they present no clinical symptoms, and their biochemical alterations show an elevation of fT4 levels, while fT3 levels are 1.11 times below the upper limit of the assay.

## 1. Introduction

First described in 1967 by Refetoff et al. [1], resistance to thyroid hormone (RTH) is a syndrome defined by elevated serum T4 (thyroxine) and, to a lesser degree, T3 (triiodothyronine), with non-suppressed thyroid-stimulating hormone (TSH) serum levels. Clinical manifestations are not specific and vary from one patient to the other (goiter, hyperactivity, developmental delay, sinus tachycardia, etc.) [2]. The discovery of discrepant biological results usually prompts further studies to establish a diagnosis.

The molecular basis of the syndrome was discovered in 1989 with the identification of a pathogenic variant (NM_000461.5:c.1033G > C p.Gly345Arg) in the thyroid hormone receptor β gene (*THRB*) [3]. More than 600 families and at least 219 mutations, mostly substitutions, have been described, with an estimated frequency of 1 case per 40,000 live births [2]. Pathogenic variants of this gene impair the receptor ability to bind T3, release co-repressors, and/or recruit co-activators, which prevents the positive or negative transactivation of target genes [2]. Inheritance is autosomal dominant, except for one family with consanguinity that had a homozygous deletion of the *THRB* gene [2,4]. Another thyroid hormone receptor, TRα, is encoded by the *THRA* gene. The relative distribution of both thyroid receptors, TRα and TRβ, varies among tissues and during development. The liver and the pituitary mostly express TRβ, while TRα is predominantly expressed in the brain, heart, bones, and digestive tract. This distribution explains the impaired feedback loop of thyroid hormone (TH) to TSH secretion when TRβ is non-functional, and the symptoms generally found in those individuals due to functional TRα include tachycardia, hyperactivity, diarrhea [5,6]. Even if more than 80% of RTH cases are due to mutations in the *THRB* gene, around 15% are due to an unknown mechanism named “non-TR-RTH” (meaning RTH syndromes that are not caused by a pathogenic variant of the TH receptors) [2]. Since its first description [7,8], the potential involvement of abnormal nuclear co-activators or co-repressors and, possibly, of *THRB* mosaicism [9], has been suspected [10,11].

Unsuppressed TSH with elevated T4/T3 could be found in other situations, such as in TSH-secreting pituitary adenomas or when other potential confounding factors are present, such as drugs (e.g., thyroxine, amiodarone). Laboratory artefacts in TH immunoassays should be looked for, especially when facing discordance between clinical and laboratory findings. TSH and TH assay interference by human anti-animal antibodies (HAA) may result in falsely normal or elevated values due to an interaction with the assay antibodies and thus mask or mimic thyroid pathologies. Other antibodies such as T4 and T3 autoantibodies, anti-streptavidin, anti-ruthenium antibodies, or substances such as heparin and biotin [12,13,14,15] may also interfere with immunoassays. Furthermore, TH serum concentration can be modified by alteration of TH-binding proteins in the absence of thyroid dysfunction. TH are hydrophobic and require binding proteins to be distributed throughout the body. The three main serum TH-binding proteins are thyroxine binding globulin (TBG), transthyretin (TTR), and albumin (ALB). When the affinity of TH for TH-binding proteins is altered, the immunoassays used to quantify free TH levels could be false. The real estimation of free TH needs to be done by direct methods, equilibrium dialysis, or ultrafiltration, which separate the small amount of free hormone from the protein-bound pool. These techniques are technically inconvenient, expensive, and only available in few laboratories [16]. The prevalence of genetic variants of TBG or TTR that change TH serum concentration is unknown [17]. TBG excess causes an elevation of total T4 but not of free T4 [12,17]. *TTR* gene mutations are subdivided into amyloidogenic and non-amyloidogenic; T4 affinity is not related to this subdivision [17]. At least 70 variants of *TTR* have been described, but only 4 have an increased T4-binding affinity (T139M (c.416C > T), G26S (c.76G > A), A129T (c.385G > A), and A129V (c.386C > T)) and are thus potentially responsible for euthyroid hyperthyroxinemia [17].

Due to variants of the gene coding for albumin (*ALB*), Familial Dysalbuminemic Hyperthyroxinemia (FDH) is the most common cause of inherited euthyroid hyperthyroxinemia in the Caucasian population, with an estimated prevalence of 1 in 10,000 individuals [17,18]. The prevalence is higher in subjects of Hispanic origin (1.0%–1.8%) than in other populations in countries such as Venezuela (0.17%), France (0.08%), and Denmark (0.01%) and is extremely rare in Japan [18]. The R218H (c.725G > A) variant, with an increased affinity for T4, is the most common variant responsible for FDH [17]. Other common variants, found in exon 7, include R218P (c.725G > C), R218S (c.724C > A), and R222I (c.737G > T) [17,18]. Rare albumin variants have been shown to increase the affinity for T3 and not T4, such as L66P (c.269T > C), which is found in exon 3 [17,18]. FDH does not result in thyroid dysfunction but, depending on the used assay, can alter serum TH levels (free T4 (fT4) and, to a lesser extent, free T3 (fT3)). Misdiagnosis can lead to an inappropriate ablative or medical treatment [17,19].

The goal of this study was to characterize the population addressed to our center for RTH to estimate the proportion of FDH and find criteria for suspected immunoassay interference.

## 2. Materials and Methods

### 2.1. Patients

Over the period from 2000 to 2015, 394 molecular analyses for RTH were sent to our reference center for rare thyroid disease at Angers University Hospital, France. They comprised 394 patients, 154 men and 240 women, who belonged to 303 different families (i.e., 303 index cases and 91 family members). The information included patients’ age, sex, clinical symptoms, fT3, fT4, and TSH levels, but not the type of TH assay used. Informed consent for genetic analysis was obtained from all patients or their legal guardians. The database was declared to the French National Data Protection Authority (CNIL) and registered under No. 900176.

### 2.2. THRB and ALB Gene Mutation

Genomic DNA was obtained from peripheral blood leucocytes, and genotyping was conducted using direct sequencing. For index cases, the germline *THRB* variants in 10 exons (NM_000461.4) and the germline *ALB* variants in exon 7 were analyzed (NM_000477.6). For screened relatives, only the presence or absence of the pathogenic variant known in the index case was searched through Sanger sequencing.

The variants were classified following the recommendation of the American College of Medical Genetics and Genomics (ACMG) [20]. ACMG classification, GnomAD data population database [21], and bioinformatics prediction tools were obtained through the use of the VarSome variant search engine [22]. Eight bioinformatics tools were used to predict the functional impact of variants such as DANN, FATHMM, FATHMM-MKL, LRT, MutationTaster, PROVEAN, SIFT, and MutationAssessor. We will use “variant” as a neutral term, “pathogenic variant” for variants affecting TRβ function, and “variant of unknown significance” (VUS) for variants for which a functional effect is not well defined.

### 2.3. Hormonal Assays and Clinical Information

Immunoassays were performed outside our center. We collected thyroid function tests, including fT4 and fT3 results, for the different assays. The reference intervals used were those provided by the manufacturer or the laboratory. To be able to compare the different assays used, we expressed the results as multiples of the upper limit (UL) of the reference range for each test. We were then able to calculate the mean and standard deviation for each parameter (fT3, fT4). We did not have the complete information on which analytical interferences had been ruled out before the blood samples were addressed to our center for *THRB* sequencing. Clinical information (goiter, hyperactivity, sinus tachycardia, etc.) was provided by the patients’ physicians. To aid the statistical analysis, we chose to keep two variables for clinical symptoms: one for the presence or absence of goiter and one for the presence or absence of other signs of hyperthyroidism. From the 394 patients, we kept 114 patients for clinical data and 122 for hormonal data. Indeed, we had 167 patients for whom possible analytical interference could not be ruled out (23 could not be tested for *ALB* variant, and 144 had no *ALB* or *THRB* variant (non FDH/RTHβ)). Furthermore, for clinical and hormonal data, we did not consider, respectively, 113 and 105 patients due to intercurrent pathologies and/or treatments or to the lack of clinical and biological data.

### 2.4. Statistical Analysis

#### 2.4.1. Univariate Analysis

Statistical analyses and graphs were made using the XLSTAT^®^ software. Comparisons of hormonal assay results between two groups were made using the non-parametrical bilateral Mann–Whitney test. When multiple groups were compared together, we used the non-parametrical Kruskal–Wallis test. A chi-squared test was also used to compare qualitative traits between groups, and appropriate corrections (Yates, Fisher’s exact test) were used whenever necessary. A Benjamini–Hochberg correction for multiple statistical testing with a false discovery rate of 5% was used.

#### 2.4.2. Multivariate Analysis

We used Python (v3.6) programming language and scikit-learn (v0.20) package, as well as the WEKA (v3.8) (Waikato Environment for Knowledge Analysis) machine learning software for multivariate analysis. To produce decision trees, we used CART and J48 (WEKA implementation of C4.5) and to produce decision rules, we used PART and JRIP (WEKA implementation of Ripper). Firstly, we split the dataset between a training set and a test set on a 65/35 basis using random stratified sampling. We then trained multiple models on the training set and optimized the hyperparameters of the models using ten-fold cross-validation on the training set. We chose the most promising model and evaluated it on the test set to get the Positive Predictive Value (PPV), Negative Predictive Value (NPV), sensitivity (SEN), and specificity (SPE) of the model for RTHβ detection. To generate the model, we also used a cost-sensitive classification to minimize the number of false positives.

## 3. Results

### 3.1. Pathogenic Variants and VUS in THRB Gene

**Figure 1:**
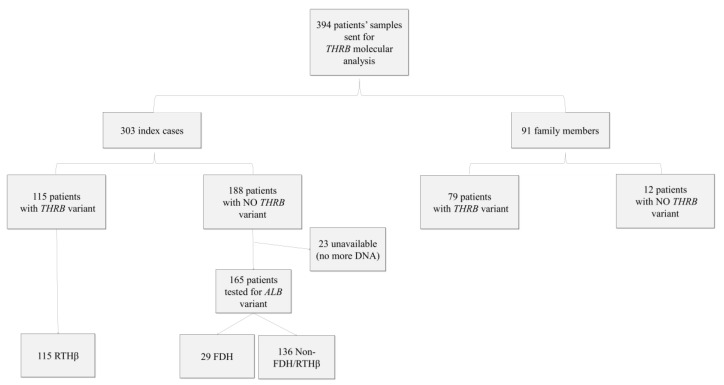
Molecular analysis performed on samples received for suspected impaired sensitivity to TH or family studies in the Reference center for rare thyroid disease of Angers University Hospital, France, between 2000 and 2015. Family members: family members related to index cases; unavailable (no DNA for *ALB* molecular analysis).

**Figure 2:**
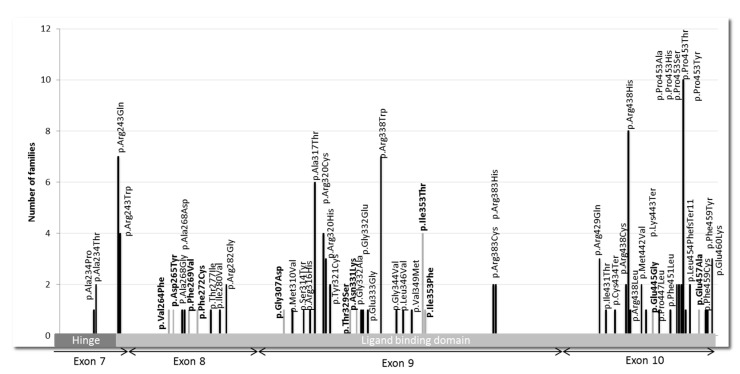
Location of 56 variants in the *THRB* gene and number of families for each variant. The *x*-axis indicates the consecutive amino acid numbers, exon, and functional domains of *THRB* gene. The *y*-axis indicates the numbers of families. Plain bars represent known variants, while grey bars represent variants with unknown significance.

Over the period from 2000 to 2015, 394 tests were carried out, of which 303 concerned index cases and 91 family studies (Table 1 and Figure 1). We found that 38% of the index cases (i.e., 115 of the 303 index cases) and 87% of the next of kin (i.e., 79 of 91) tested were carriers of pathogenic *THRB* variants or VUS. We found 56 different variants in the *THRB* gene, located in the ligand-binding domain (LBD) and its adjacent hinge region (Figure 2). Of these, 45 variants were known as pathogenic, and 11 were new findings.

We found 45 *THRB* pathogenic variants that were mostly missense variants, except for 3 that resulted in a stop codon. The 5 most frequent mutations among the 115 found in our center were P453T (c.1357C > A) with 10 index cases, i.e., 9%; R438H (c.1313G > A) with 8 index cases, i.e., 7%; R243Q (c.728G > A) with 7 index cases, i.e., 6%; R338W (c.1012C > T) with 7 index cases, i.e., 6.1%; and A317T (c.949G > A) with 6 index cases, i.e., 5.2% (Figure 2).

We identified 11 new *THRB* variants across 14 index cases that were not described in variant databases. We used consensus recommendation from the ACMG based on various criteria, such as population data, computational and functional data, as well as segregation to classify the variants. The 11 missense variants could be classified as likely pathogenic (Table 2).

### 3.2. Patients with FDH

Among the 188 index cases without detected variants in *THRB*, we searched for the most frequent variants found in FDH in 165 index cases (23 exhausted DNA samples). The R218H (c.725G >A) variant of the *ALB* gene was found in 29 cases (18%). No other variants in exon 7 of the *ALB* gene were found. Capillary electrophoresis of serum proteins was normal.

### 3.3. Patients without FDH or RTHβ (Non FDH/RTHβ)

From the 394 patients, we had 144 patients that did not have a variant of *THRB* or a variant of *ALB*. These patients tended to be older and were mostly isolated cases, with a higher proportion of women than in the FDH or RTHβ groups (Table 1). These patients were not considered for clinical and hormonal statistical analyses.

### 3.4. Clinical Information

We were able to retrieve clinical data for 114 patients: 97 of them carried *THRB* variants (RTHβ group), and 17 carried *ALB* variants (FDH group). Of these patients, 60% had a goiter (assessed through echography), and 61% had signs of hyperthyroidism (mostly tachycardia, nervousness, hyperactivity).

**Figure 3:**
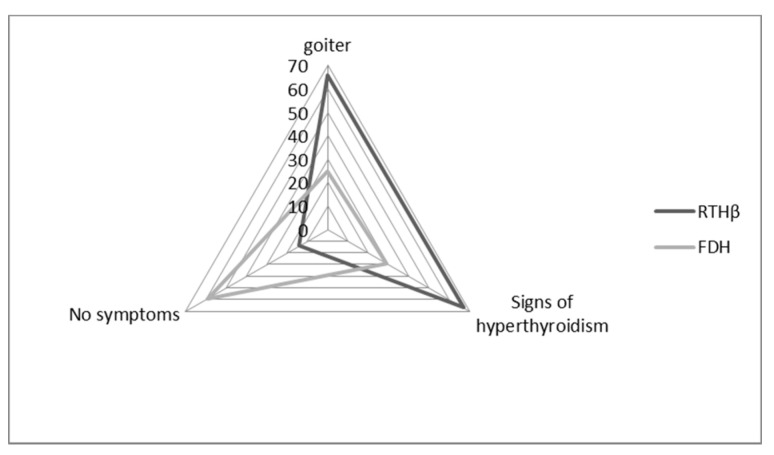
Distribution of the different clinical features among patient with RTHβ and FDH. Radar plot showing the proportion of patients (%) with each clinical feature: goiter, signs of hyperthyroidism, or asymptomatic.

In the FDH group, patients were more frequently asymptomatic (59%) when compared to the RTHβ group (14%, *p* = 0.0002) (Figure 3). In the FDH group, there were significantly fewer symptoms of hyperthyroidism (29%) than in the RTHβ group (67%, *p* = 0.007) (Figure 3). On the other hand, goiter was statistically less frequent in the FDH group (25%) compared to the RTHβ group (66%) (*p* = 0.004).

### 3.5. Hormone Assays

Regarding the hormonal assays, we obtained information for 122 subjects: 105 patients carried *THRB* variants (RTHβ group), and 17 carried *ALB* variants (FDH group).

Regarding all available hormonal results, 82% of the patients had both elevated fT3 and fT4, 15% presented an isolated increase of fT4, and 3% an isolated increase of fT3.

**Figure 4:**
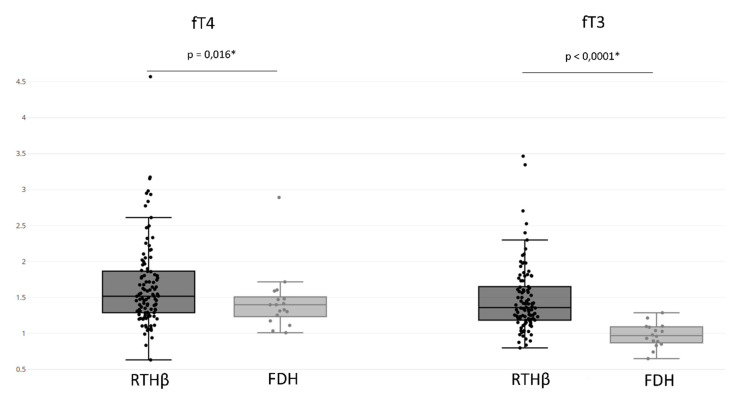
Thyroid function assays (free T4 (fT4) and free T3 (fT3) levels) in several patients with RTHβ or FDH. fT4 and fT3 levels are expressed as multiples of the upper limit of the assay. The * symbol indicates a statistically significant *p*-value after correction for multiple statistical tests (*p* < 0.05).

The RTHβ group presented an fT4 value of 1.669 ± 0.591 UL and an fT3 value of 1.482 ± 0.470 UL (Figure 4). In addition, 89% of patients in the RTHβ group had both increased fT4 and fT3, and rarely we observed an isolated increase of fT4 (8%) or fT3 (3%) (Table 3). We also compared the fT3 and fT4 levels between patients with the five most frequent pathogenic variants, having at least five patients with known hormonal results: P453T (seven values), R438H (six values), R243Q (six values), R243W (five values), R383H (five values), E460K (five values), and A317T (five values). We found no differences for fT4 (*p* = 0.122) or fT3 (*p* = 0.55) levels.

The FDH group had an fT4 value of 1.442 ± 0.422 UL and an fT3 value of 0.975 ± 0.167 UL (Figure 4). When comparing the FDH group with the RTHβ group, the RTHβ group had statistically significant higher values of both fT3 (*p*-value < 0.0001) and fT4 (*p*-value = 0.016). We found that 44% of patients with FDH presented increased fT4 and fT3 levels, with more having an isolated increase of fT4 (56%). However, there were no patients with isolated increases of fT3. This isolated elevation of fT4 was statistically more often found compared to the RTHβ group (*p*-value < 0.0001) (Table 3).

### 3.6. Multivariate Model to Differentiate between THRB and ALB Variants

The goal of our multivariate analysis was to find if a simple algorithm could help physicians distinguish between “true” RTHβ and FDH. We considered only patients without any missing values for the analysis. This multivariate approach models a general tendency across different TH assays on which FDH has a variable impact. After random stratified sampling, 58 patients were assigned to the training set and 32 to the test set. Based on the training set, we chose a model delivered by the JRIP algorithm, which creates rules. The rules were as follow:

• If a patient’s fT3 is above 1.11, then he/she has a THRB variant.
• For all the other cases, the patient has FDH.

On the test set (Table 4), our model made correct predictions for 26/32 patients (81% of the test set). This model had a very good SPE of 80%, with a PPV of 96% and a SEN of 81% for the prediction of RTHβ due to a *THRB* variant. However, it could only get an NPV of 44%. It had a Receiver Operating Characteristic Area Under Curve of 0.8.

## 4. Discussion

In our study objectives, we wanted to characterize the landscape of French patients with dissociated results for thyroid tests (unsuppressed TSH and elevated fT4 and/or fT3), as well as find parameters that could help us to differentiate causes of the discrepancy.

One-third (115 families of a total 303) of our sequencing demands for suspected RTH resulted in the discovery of a *THRB* gene variant. Regarding the different mutations found in the *THRB* gene, the most frequent were R243Q, A317T, R338W, R438H, and P453T, as already described [20]. Almost two-thirds of our sequencing demands (188 families of a total 303) for suspected RTH had no pathogenic *THRB* gene variant. Because only approximately 15% of the subjects with RTH do not have a *THRB* variant based on the literature [2], it was suspected that a large proportion of these discrepant results in thyroid function tests would reveal causes other than a true RTH.

We found that 18% of patients without a *THRB* variant harbored the most frequent variant of the *ALB* gene (R218H (c.725G > A)). To our knowledge, this is the first time that a study has evaluated the proportion of FDH among patients with unsuppressed TSH and elevated fT4 and fT3 in France. Secondly, in multiple cases/families (136 non-FDH/RTHβ), no explanation for their abnormal biological findings could be identified. This is suggestive of other analytical interferences.

We found that patients with FDH tended to have lower levels of fT4 and fT3 compared to the RTHβ group. They also more often had an isolated elevation of fT4 which was expected. We tried to create a simple yet potentially effective model to help physicians choose whether to prescribe an expensive molecular analysis and found that if the fT3 level is not clearly elevated (<1.11 times the upper limit of the test), then it is highly unlikely that a patient has RTHβ. Interestingly, our model did not use clinical variables such as goiter, showing that these variables were not as reliable predictors of RTHβ versus FDH as the fT3 levels. These results are coherent with the fact that FDH had already been described to yield artificially increased fT4 levels and are in agreement with in vitro assay results, since the variant R218H of the human serum albumin leads to an increased affinity for T4 but not T3, explaining the preferential interference in fT4 assays [17,18]. It largely depends on the technique used but does not necessarily correlate with the usage of one- or two-step methods, even if, in theory, the one-step method is more impacted by this interference [23,24]. Also, other hormonal assays, when available, may aid in the differential diagnosis of RTHβ versus FDH (for example total T3 is elevated in RTHβ but rarely in FDH). Clinically, patients with an *ALB* variant have less often a goiter or other clinical symptoms compared to patients with a *THRB* variant and generally tend to be more often asymptomatic compared to the other groups. In conclusion, an elevation of fT4 in the presence of levels of fT3 that are normal or less than 1.11 times the upper limit of the assay and combined with no clinical symptoms is in favor of FDH.

For patients with no *THRB* or *ALB* variants found, another etiology must be searched to explain their clinical and biological profile. Most of our patients were subjected to MRI of the pituitary, to absolutely rule out a TSH-secreting pituitary adenoma, which is one of the possible differential diagnoses of RTHβ. Further tests such as TSH stimulation by TRH or suppression by somatostatin analogs were sometimes necessary to eliminate the presence of a microadenoma. Also, there is a possibility that many patients in this group had non-thyroid diseases, associated or not with analytical artefacts that may have rendered the clinical presentation more difficult to assess properly and thus led to this genetic search. Interferences are very diverse and for some of them solutions exist. In fact, antibody-based interference may be filtered out with PEG precipitation, and interference due to substances like biotin or heparin should be systematically searched for through a careful analysis. Most of the time, only the physicians have access to biological and clinical information regarding patients. Hence, the easiest way of getting rid of this interference is to use at least two assays based on different techniques [15,23,25] and to interpret the results together with the clinical features. Finally, other genes modifying the TH action could be involved in discrepant thyroid function tests. It is probable that some patients in this group had a real RTH without a variant of *THRB* (a.k.a., “non-TR-RTH”). Exploring family cases and patients particularly symptomatic through extensive genetic search (whole-exome and whole-genome sequencing) may lead us toward understanding the genetic basis behind “non-TR-RTH”.

A limit of this study regards the diverse origins of the hormonal assays. They came from several different laboratories and were performed with many different techniques, that are variably impacted by each interference [13,14,15,23,24,26].

## 5. Conclusions

Our study shows that clinical and laboratory findings should always be carefully interpreted together to determine if interference is the most likely explanation for the results of hormonal assays. FDH may be variably detected by different hormonal assays. Taking these elements into account, we found that, in patients suspected of having RTH without the presence of a *THRB* variant, their abnormal hormonal assay was due in 18% of the cases to an *ALB* variant responsible for FDH. Patients with FDH often have no clinical symptoms and elevated fT4 levels, with normal or mildly elevated fT3 levels, which appear 1.11 times below the upper limit compared to patients with a *THRB* variant. In case of suspected RTH, we first recommend repeating the assay with a different technique and eliminating analytical interference as much as possible, especially in case of discordance between the biological data and the clinical presentation. Given the higher prevalence of FDH in the general population with respect to RTH, itself more prevalent than TSH-secreting pituitary adenomas, we recommend to then search for an *ALB* variant (as well as other variants of TH transport proteins), and sequence the *THRB* gene, which will avert submitting many patients to unnecessary and costly MRI. This strategy would help avoid misdiagnoses and/or mistreatments.

## Figures and Tables

**Table 1:** Age, sex, number of families/isolated cases of patients with suspected thyroid hormone (RTH) variants.

	ALL	RTHβ	FDH	Non-FDH/RTHβ	Not Available
Age (years)	32.3 ± 20.9	29.4 ± 21.2	28.5 ± 21.5	37.9 ± 19.2	37.8 ± 12.6.
Males (%)	39	43	48	33	22
Index cases	303	115	29	136	23
Next of kin	91	79	4	8	0

ALL (all patients referred to our center); RTHβ (resistance to thyroid hormone with *THRB* variant); FDH (Familial Dysalbuminemic Hyperthyroxinemia with *ALB* variant); non-FDH/RTHβ (patient with no *THRB* or *ALB* variant); not available (no DNA for *ALB* molecular analysis); Next of kin: family members related to index cases.

**Table 2:** Patients with RTH carrying newly described *THRB* germline variants. Variant features, population database report, in silico predictions of pathogenicity, as well as American College of Medical Genetics and Genomics (ACMG) classification of the newly described *THRB* variants.

EXON	HGVSc^1^ NM_000461.4	HGVSp^2^ NP_000452.2	RS ID	gnomAD	Families	Patients	In Silico Predictions Algorithms	Classification Following ACMG
8	c.790G > T	p.(Val264Phe)	rs1559415493	0	1	1	7 pathogenic vs. 1 benign *	Likely pathogenic (PM1, PM2, PP2, PP3)
8	c.793G > T	p.(Asp265Tyr)	none	0	1	1	7 pathogenic vs. 1 benign *	Likely pathogenic (PM1, PM2, PP2, PP3)
8	c.805T > G	p.(Phe269Val)	none	0	1	2	8 pathogenic **	Likely pathogenic (PM1, PM2, PP2, PP3)
8	c.815T > G	p.(Phe272Cys)	none	0	1	4	7 pathogenic vs. 1 benign *	Likely pathogenic (PM1, PM2, PP2, PP3)
9	c.920G > A	p.(Gly307Asp)	rs1553611101	0	1	1	8 pathogenic **	Likely pathogenic (PM1, PM2, PP2, PP3)
9	c.985A > T	p(.Thr329Ser)	none	0	1	1	7 pathogenic vs. 1 benign ***	Likely pathogenic (PM1, PM2, PP2, PP3)
9	c.993T > A	p.(Asn331Lys)	none	0	1	1	6 pathogenic vs. 2 benign ****	Likely pathogenic (PM1, PM2, PP2, PP3, PM5)
9	c.1058T > C	p.(Ile353Thr)	none	0	4	7	8 pathogenic **	Likely pathogenic (PM1, PM2, PP2, PP3)
9	c.1057A > T	p.(Ile353Phe)	none	0	1	1	8 pathogenic **	Likely pathogenic (PM1, PM2, PP2, PP3)
10	c.1334A > G	p.(Glu445Gly)	none	0	1	2	8 pathogenic **	Likely pathogenic (PM1, PM2, PP2, PP3)
10	c.1370A > C *****	p.(Glu457Ala) *****	none	0	1	1	8 pathogenic **	Likely pathogenic (PM1, PM2, PP2, PP3)

In gray, figures variants found in our center, already published in “Annales d’Endocrinologie”, a French journal, but not found in Pubmed (C. Bournaud, F. Savagner, F. Borson-Chazot, O. Revol, M. Oliel, S. Achard, Y. Malthiery, J. Orgiazzi, P. Rodien, Annales d’Endocrinologie Vol 66, N° 5, octobre 2005 p. 505 Doi:AE-10-2005-66-5-0003-4266-101019-200505896 and H. Combe, I. Khochtali, H. Bihan, P. Rodien, R. Cohen, F. Savagner, Annales d’Endocrinologie Vol 66, N° 5, octobre 2005 pp. 505–506 Doi:AE-10-2005-66-5-0003-4266-101019-200505897). Two variants had an already existing RS ID but without associated publication(s). ^1^ Human Genome Variation Society DNA nomenclature. ^2^ Human Genome Variation Society protein nomenclature. * Seven pathogenic predictions from DANN, FATHMM, LRT, MutationTaster, PROVEAN, FATHMM-MKL, and SIFT vs. one benign prediction from MutationAssessor. ** Eight pathogenic predictions from DANN, FATHMM, LRT, MutationAssessor, MutationTaster, PROVEAN, FATHMM-MKL, and SIFT. *** Seven pathogenic predictions from DANN, FATHMM, MutationAssessor, MutationTaster, PROVEAN, FATHMM-MKL, and SIFT vs. one benign prediction from LRT. **** Six pathogenic predictions from DANN, FATHMM, LRT, MutationTaster, FATHMM-MKL, and SIFT vs. two benign predictions from MutationAssessor and PROVEAN NA: Not Applicable. ***** Variant already described in an abstract (Chatterjee K, Adams M, Collingwood T, Matthews C, Rajanayagam O. “Functional analysis of mutant thyroid hormone receptors in thyroid hormone resistance syndrome”, Second International Workshop on Thyroid Hormone Resistance, 1995; Padua, Italy) but not indexed in Medline nor found in Pubmed. PM1: Located in a mutational hot spot and/or critical and well-established functional domain (e.g., active site of an enzyme) without benign variation. PM2: Absent from controls (or at extremely low frequency if recessive) in Exome Sequencing Project, 1000 Genomes Project, or Exome Aggregation Consortium. PP2: Missense variant in a gene that has a low rate of benign missense variation and in which missense variants are a common mechanism of disease. PP3: Multiple lines of computational evidence support a deleterious effect on the gene or gene product (conservation, evolutionary, splicing impact, etc.). PM5: Novel missense change at an amino acid residue where a different missense change determined to be pathogenic has been seen before.

**Table 3:** Percentage of index cases with variations of fT3 and fT4 according to the diagnosis of RTHβ or FDH.

	fT4 ↗ fT3 ↗	fT4 ↗ fT3 Nl	fT4 Nl fT3 ↗
RTHβ	89%	8%	3%
FDH	44%	56%	0%

Nl: ft4 or fT3 is considered normal for each assay **↗**: ft4 or fT3 above the upper limit of each assay.

**Table 4:** Results of the multivariate model on the test set. It was trained to predict whether or not a patient had a pathogenic *THRB* variant based on clinical and biological features. A positive prediction by the model refers to the prediction of a patient having a pathogenic *THRB* variant.

		*Genetic Variant*	
		*THRB*	*ALB*
*Model prediction about the presence of a THRB variant*	*positive*	*22*	*1*
	*negative*	*5*	*4*
